# Seasonal Fe Uptake of Young Citrus Trees and Its Contribution to the Development of New Organs

**DOI:** 10.3390/plants10010079

**Published:** 2021-01-02

**Authors:** Mary-Rus Martínez-Cuenca, Belen Martínez-Alcántara, Jorge Millos, Francisco Legaz, Ana Quiñones

**Affiliations:** 1 Department of Citriculture and Vegetal Production, Valencian Institute for Agricultural Research–IVIA, Crta. CV-315, 46113 Moncada, Valencia, Spain; martinez_belalc@gva.es (B.M.-A.); legaz.fra@gmail.com (F.L.); 2 Service of Food Security and Sustainable Development-C.A.C.T.I., Vigo University, 36310 Vigo, Pontevedra, Spain; jmillos@uvigo.es; 3 Center for the Development of Sustainable Agriculture-CDAS, Valencian Institute for Agricultural Research–IVIA, Crta. CV-315, 46113 Moncada, Valencia, Spain

**Keywords:** iron, ^57^Fe, *Citrus clementina*, enrichment, isotope, mineral transport

## Abstract

This work quantifies Fe uptake in young citrus trees, its partitioning among plant compartments, and the contribution of the Fe absorbed from fertilizer to the development of new tissues. A soil pot experiment was conducted using 4-year-old clementine trees (*Citrus clementina* Hort ex Tan), and a dose of 240 mg Fe was applied by labeled fertilizer (92% atom ^57^Fe excess). Plants were uprooted at five different phenologic states: end of flowering (May 15), end of fruit setting and fruit drop (July 1), two fruit growing moments (August 1 and October 15), and at complete fruit maturity (December 10). The Fe accumulated in the root system exceeded 90% of the total Fe content in the plant. All organs progressively enriched with ^57^Fe (8.5–15.5% and 7.4–9.9% for young and old organs, respectively). Reproductive ones reached the highest increase (111% between May and October). ^57^Fe enrichment from woody organs reflects an increasing gradient to sink organs. The root system accumulated 80% of the Fe absorbed from the fertilizer, but the young organs accumulated relatively more Fe uptake during flowering and fruit setting (15.6% and 13.8%, respectively) than old organs (around 9.8%). Although iron derived from fertilizer (Fedff) preferably supplied young organs (16.7–31.0%) against old ones (2.5–14.9%), it only represented between 13.8% and 21.4% of its content. The use efficiency of the applied Fe (FeUE) barely exceeded 15%. The lowest FeUE were found in young and old organs of the aerial part (1.1–1.8% and 0.7–1.2%, respectively). Since the pattern of the seasonal absorption of Fe is similar to the monthly distribution curve of the supplied Fe, it is recommended that the application of Fe chelates in calcareous soils should be performed in a similar way to that proposed in this curve.

## 1. Introduction

Although most soils contain enough iron (Fe) to satisfy the needs of crops, in the presence of oxygen, an alkaline medium, and an excess of soil moisture, this ion precipitates as the insoluble hydroxide, which is a scarcely soluble Fe form that, therefore, causes serious problems of iron chlorosis for plants. Thus, in alkaline soils such as those in the Mediterranean basin, high levels of bicarbonate ions are the main cause of iron chlorosis in citrus plantations. The most effective way to combat this deficiency is by applying iron chelates. However, their use markedly increases operating costs, accounting for 14% of the total cost of fertilization [1]. 

The importance of Fe in plants is well known, as it is involved in a series of metabolic processes that are fundamental to their development. This is the case of photosynthesis, since Fe participates in several of the steps of the chlorophyll biosynthetic pathway regulating the activity of the enzyme system for the formation of protochlorophyll and other pigments, besides being responsible for the morphology, structure, and maintenance of chloroplasts [2]. So, the deficiency of Fe impairs the photosynthetic capacity of the tree decreasing photoassimilated compounds such as sugar levels and starch. This ion is also part of a good number of enzyme systems important for the metabolism of the plants, such as the protein ferredoxin that acts as a final acceptor of electrons and whose high redox potential allows to reduce substances as NADP^+^, nitrate, oxygen, and sulfate [2,3]. Finally, free Fe can interact with oxygen to form superoxide anions, which damage membranes by degrading unsaturated lipid components, especially in the leaf. Excess of Fe is stored in an Fe-protein complex called phytoferritin, which contains between 5400 and 6200 atoms of Fe (III) as a ferric oxide-phosphate complex [4]. Therefore, Fe deficiency (or Fe chlorosis) is one of the main abiotic stresses in the citrus and fruit trees of the Mediterranean basin, mainly as a consequence of the predominance of calcareous soils. Although the response may vary from one species to another, iron chlorosis reduces the longevity of the plantation, as it affects the development of new shoots due to the low translocation of the element from the adult leaves to the new tissues. Therefore, the vegetative shoots are less vigorous, and their leaves are smaller, even giving rise to an early defoliation of the shoots and their progressive death from the apical zone. Fruit production and quality are also reduced, and at levels of acute deficiency, mature fruits are usually smaller in size, with soft skin and lack of color. The flavedo of oranges and mandarins characteristically acquire a yellowish color, without reaching the orange-reddish tonalities of the normal fruits, and it diminishes the content of soluble solids in the juice [5].

As a dicotyledonous plant, citrus develop a strategy I mechanism for mobilization and acquisition of Fe from the soil or soil solution [6,7]. Regarding the mobility of Fe in the plant, it is known that this hardly remobilizes from the old leaves to the young developing tissues, since the Fe is transported from the roots to the aerial part via xylem, to the cells of the mesophyll mainly bound to the citrate ion. Nevertheless, once fixed in the aerial organs, Fe remains immobile due to its retention in organic acids (mainly citric and malic) of the foliar parenchyma tissue. Thus, leaves accumulate Fe as they age and do not remobilize it to new shoots. This fact was confirmed when applying 3 doses of Fe via foliar (Rayplex) and soil (Fe-EDDHA) to orange trees of the "Navelina" variety cultivated in a calcareous soil and with flood irrigation [8]. The Fe applied on the leaves of the spring flush did not translocate to the shoots of the following seasonal flushes of the same year, or to the fruits undergoing development. At the next vegetative cycle, the leaves of the spring flush of the treated trees and the control leaves showed identical Fe concentrations. In addition, the foliar levels increased correlatively to the dose applied by foliar treatment. This confirms the low mobility of this nutrient between leaf tissues, at least in citrus trees. However, we have no information on its translocation from the woody organs (old branches, trunk, and root system) to the new tissues.

Several factors are involved in the absorption of Fe. In addition to those intrinsic to the plant (vigor of the variety and pattern and age), other examples include the culture medium (calcareous soils rich in bicarbonate ions and with low pH that cause an excessive release of ion phosphate and other metals), as well as irrigation management and fertilization practices. Despite the importance of this latter, few studies have focused on Fe dose, its time of application or seasonal distribution, and Fe fractionation (number of applications) in citrus.

The Fe dose depends on the characteristics of the plantation (tree size, variety, rootstock, plantation frame, production, soil type, cropping system, etc.), as well as the nutritional status of the plant evaluated by leaf analysis. However, there are a few studies on the response of citrus fruits to differential doses of Fe. The application of two doses of Fe-EDDHA to adult mandarin trees increased the total leaf chlorophyll content and improved their yield, with an increase in the number of fruit sets [9]. The lower dose (24 kg ha^−1^) could be considered the most convenient from a nutritional and economic point of view.

The time of application of nutrients is also important, as it is known that these are absorbed by citrus throughout the year but not steadily throughout the cycle. In general, the time of maximum absorption includes the end of spring, summer, and early fall, and the minimum during winter. The decrease in the absorption at very low temperatures seems to be due to a lower fluidity in the cytoplasmic membrane and, therefore, to a greater resistance to the passage of the ions. Moreover, the growth of the fibrous roots is also considerably reduced. Recent studies have established the distribution pattern of nitrogen fertilizer in citrus throughout the vegetative cycle of the plant [10], but similar information about Fe is not currently available.

The interest of fractionation of the Fe dose lies in the fact that it fulfills the function of maintaining a level of this micronutrient available to the plant in a constant and prolonged manner, thereby improving the efficiency of its use and reducing the fertilization costs. However, a few results on this aspect can be found in the current literature, and, in general, the studies evaluate the relative distribution between the different organs of the total accumulated Fe in the plant up to the time of extraction, chlorophyll concentration, or increase in production [8,11]. Thus, a study carried out in adult trees of the variety “Clementina de Nules” (Nules clementine), where a dose of 3 g Fe (Fe-EDDHA) was applied for 6 months (April to September) and distributed in 3, 6, 12, and 24 applications at intervals of 8, 4, 2, or 1 weeks, respectively, concluded that the application of the chelate increased chlorophyll production and concentration in the leaves [11]. In addition, these authors noticed that the best responses were achieved when the chelate was added in periods of more than 1 week. However, the use of ferric fertilizers enriched with ^57^Fe not only determines the uptake of Fe by the crop [12,13], but also its destination within the plant, in the culture medium, the seasonal absorption of Fe from the applied fertilizer, and its relative distribution among the different organs of the plant. The use of isotopes as tracers constitutes a powerful research tool that makes it possible to obtain comprehensive information about the Fe dynamics in the plant–soil system that is not accessible by the conventional procedures and techniques of study [14,15].

Finally, closely related to the dose of Fe applied is the Fe use efficiency (FeUE), which estimates the proportion of Fe absorbed by the plant in relation to the dose applied. Generally, FeUE decreases with the increase in fertilizer dose, so this parameter will be of agronomic interest as it influences the Fe consumption of the crop. A low efficiency is not always indicative of a low absorption capacity of the crop, but rather of an excessive dose. Therefore, better FeUE values will be obtained when the Fe dose is more matched to the needs of the plant, which will in turn reduce the fertilizer costs.

Specifically, the objective of the present work is to quantify the effect of the seasonal distribution of a dose of Fe on the following aspects: (a) quantification of Fe absorption along the vegetative cycle and its use efficiency; (b) distribution of Fe absorbed among the different plant organs at different phenological moments; and (c) relative contribution of the Fe absorbed from the fertilizer to the fruit setting, as well as to the vegetative development of the different flushes. The use of a chelate enriched with ^57^Fe allows them to be quantified exhaustively. In this way, this study will expand the knowledge of the Fe dynamics in plants, in order to establish the fertilizer criteria of this nutrient and to optimize its seasonal application, thus limiting the supply of excessive amounts of ferric fertilizers, which will result in a saving in production costs.

## 2. Results and Discussion

### 2.1. Biomass and Its Relative Distribution

Trees presented an average dry weight (Table 1) of 746.0 ± 92.4 and 1373.1 ± 182.5 g in the initial (end of flowering) and final extractions (December), respectively. So, plants almost doubled their biomass as a consequence of the development of reproductive structures, new shoots, and the growth of old existing organs. The greatest increases in biomass occurred in plants uprooted between July and May (24.4%) and between October and August (20.4%) due to the development of the second and third flushes, respectively. When analyzed throughout the cycle, biomass increment was particularly marked in the reproductive organs as a whole (flowers and fruit) and in fibrous roots, with values of 785.3% and 120.3%, respectively. 

In the literature, the dry weight of the whole tree is closely related to the species, the age of the tree, cultivation practices, and the soil and climate conditions in the area. Our results are similar to the values found by other authors in young tress. Nules clementine trees harvested in December obtained a biomass at dormancy of 1.25 and 1.45 kg according to whether they received an organic fertilizer of plant or animal origin, respectively [16]. Four-year-old Valencia Late trees grown in a sandy soil reached an average weight of 5.0 kg at the end of the cycle [17]. Three-year-old Lane Late trees grown in the field, obtained a biomass of 4.4 kg in at the end of the second vegetative cycle [18]. The biomass of 3-year-old Hamlin orange trees sampled in May varied from 2.3 to 5.0 kg, depending on the doses of N provided by fertigation [19]. However, dry weight biomass in adult trees can be notable different, from the low values of 38 kg found in 8-year-old Naveline orange trees [20] to values of 156 kg for 15-year-old Wilking clementine trees [21] or even around 320 kg for 20-year-old Shamouti orange trees [22].

**Figure 1:**
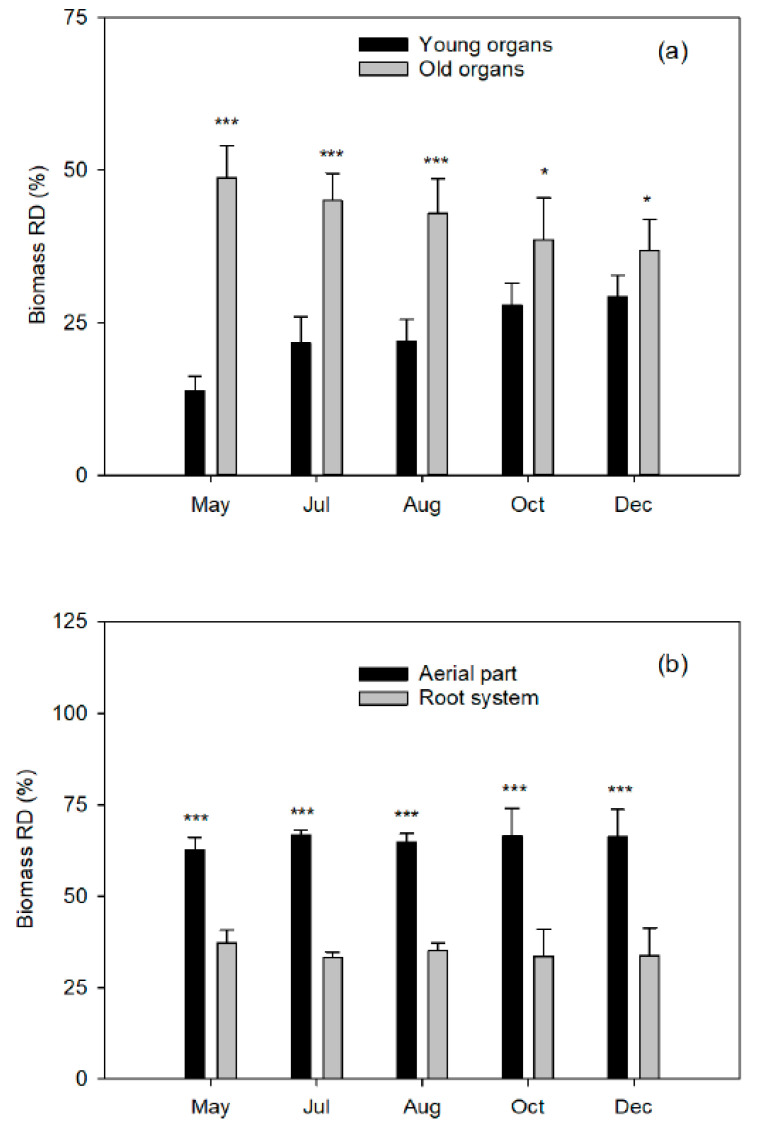
Relative distribution (%) of dry-biomass (RBD) in (**a**) young^Z^ and old^Y^ organs from the aerial part in relation to the whole plant and (**b**) total aerial part and root system^X^ of Nules clementine trees uprooted in May (flowering), July (fruit set and the end of fruit drop), August (fruit growth), October (fruit growth), and December (fruit maturity). Values are means ±SD (n = 4). Significant effects of Fe dose are given at *p*  ≤  0.05 (*) and *p*  ≤  0.001 (***) at each harvest event. ^Z^Young organs: May: flowers, fruits, and leaves and branches from spring flush; July and August: developing fruits and leaves and branches from spring and summer flushes; October: developing fruits and leaves and branches from spring, summer, and fall flushes; and December: mature fruits and leaves and branches from spring, summer, and fall flushes. ^Y^Old organs: leaves and branches from the year before flushes, branches without leaves, and trunk. ^X^Root system: coarse and fibrous roots.

Interestingly, regardless of the increase in weight recorded, the relative biomass distribution (RBD) of the aerial part and the root system in relation to the total biomass of the plant (Figure 1b) remained practically constant throughout the cycle, with mean values of 65% and 35%, respectively. Although small, an antiparallel trend can be observed in the RBD of young and old organs (increasing and decreasing, respectively) throughout the cycle (Figure 1a). The RBD of the aerial part and the root system also depends on the age of the plants and our results are in line to other experiments on young citrus trees [18,23,24]. In adult trees, however, the values of biomass accumulated in the aerial part are slightly higher than ours, with values between 65% and 85% [20,21,22,25]. The woody parts of the tree (trunk and branches) accumulate a higher percentage of biomass due to their older age (49–67%, respectively), while they represent 25–30% in young tress [17,24,26].

### 2.2. Fe Concentration

The Fe concentration in the leaves is closely related to their age (Table 2), and thus the highest values were found in the old leaves (59.2 ± 3.6 mg Fe 1000 g^−1^ DW in the May sample) and the lowest in the leaves of the fall flushes, which are the youngest (27.1 ± 5.4 mg Fe 1000 g^−1^ DW in the October sample). In addition, both leaf types presented opposite patterns in the Fe concentration throughout the cycle. While the Fe concentration in the old leaves decreased irregularly over time, presenting an average concentration of 50.5 ± 5.2 mg Fe 1000 g^−1^ DW but without any significant differences between samples, it increased progressively in the young leaves of the different flushes (23.5%, 20.6%, and 15.1% in spring, summer, and fall leaves, respectively) measured between their appearance (May, July, and October, respectively) and the end of the cycle (December). Conversely, the concentration of Fe in the young reproductive organs (flowers and fruits) decreased very sharply (from 52.2 ± 2.1 to 11.1±0.8 mg Fe 1000 g^−1^ DW, a decrease in 78.8%) from May to December as a result of the dilution of this element associated with the increase in biomass in growing fruits. The old organs (branches and trunk) maintained fairly stable Fe values throughout the cycle with average values between 35.7 ± 1.2 and 40.0 ± 3.3 mg Fe 1000 g^−1^ DW, respectively. The coarse and fibrous roots showed average Fe concentrations of 188.2 ± 26.5 and 2428.7 ± 276.5 mg Fe 1000 g^−1^ DW, respectively, which were much higher than the aerial part, especially in the latter case, and displayed an increasing trend from flowering to the end of fruit set (July) and then decreased until the end of the cycle.

This pattern in the Fe concentration in the different organs of the plant has also been seen in citrus and other crops. The relationship in the Fe concentration in *Citrus aurantium* seedlings was of 77:1:11 between root system:stem:leaves, being the value in the root of 496 µg g^−1^ DW [27], and it dropped by half under waterlogging stress and the ratio changed to 59:1:8. In kiwi [28], roots had the highest Fe concentration values, especially the fibrous roots (550 mg kg^−1^ DW), and levels in the old leaves exceeded that in new leaves (280 and 200 mg kg^−1^ DW, respectively). In peaches, while the highest accumulation of Fe occurred again in the root system (1900 mg kg^−1^ DW), the fraction of the plant with the second highest concentration was the totally expanded upper leaves (220 mg kg^−1^ DW) and finally, the stem and basal leaves, which accumulated over 100 mg kg^−1^ DW [29]. Although these authors did not observe a concentration gradient between leaf layers (apical, expanded, and basal leaves), they described a fractionation of the Fe within the leaf itself. In particular, the highest Fe concentration in the leaves is found in the main nerve and becomes especially apparent under conditions of Fe deficiency. In the same line, the immobilization of Fe in ferric form in the apoplast was described in chlorotic leaves of citrus and the low efficiency of the enzyme ferric reductase was the main cause of the symptomatology [30].

### 2.3. Fe Content and Its Relative Distribution

The Fe content (Table 3) increased progressively in all the vegetative organs between the beginning and the end of the cycle, except for old leaves. The decrease in the Fe content observed in the old leaves was due more to the loss of biomass when senescence than to the decrease in its concentration. By contrast, Fe content in the reproductive organs decreased progressively from May to August (61.1%) due mainly to the drastic decrease in Fe concentration in this period (Table 2). The scant increase in biomass of 25% between May and August (Table 1) associated with the physiological dropping of fruit in the setting process caused a reduction in 22.1% in Fe content between May and July. Towards the end of the cycle (October and December), accumulated Fe increased considerably in these organs due to the increase in biomass in developing and ripe fruits (205.9% and 130.8%, respectively), as the concentration continued to decrease in both periods (18.7% and 13.5%, respectively).

**Figure 2:**
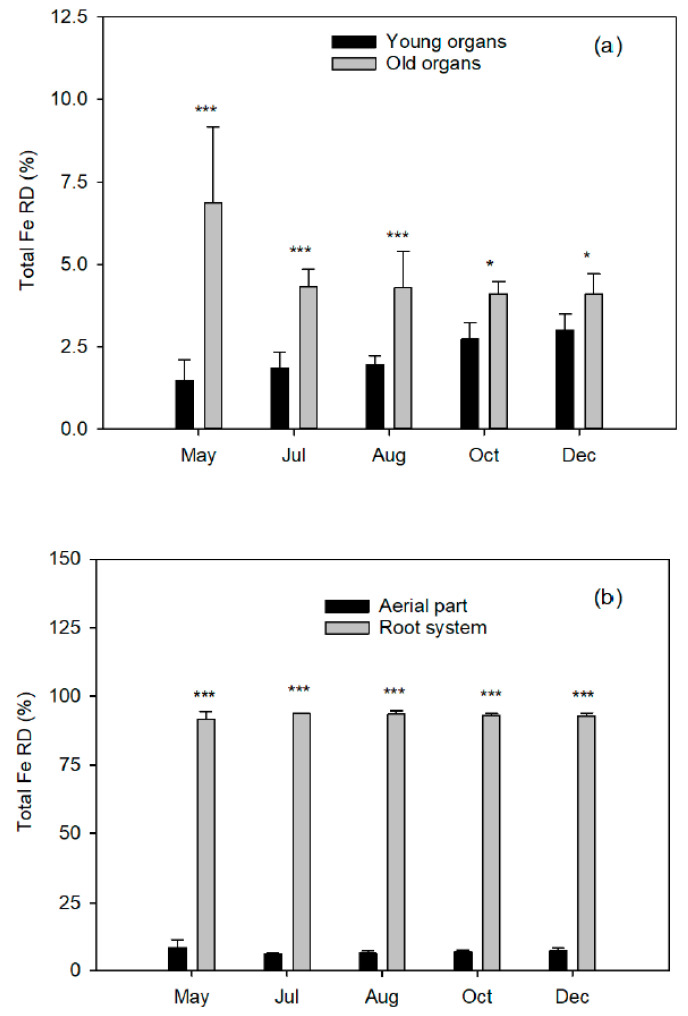
Relative distribution (%) of total iron (FeRD) in (**a**) young ^Z^ and old ^Y^ organs from the aerial part in relation to the whole plant and (**b**) aerial part and root system ^X^ of Nules clementine trees uprooted in May (flowering), July (fruit set and the end of fruit drop), August (fruit growth), October (fruit growth), and December (fruit maturity). Values are means ±SD (n=4). Significant effects of Fe dose are given at *p*  ≤  0.05 (*) and *p*  ≤  0.001 (***) at each harvest event. ^Z,Y,X:^ See Figure 1.

Aerial part only accumulated 6–8% of the total Fe content (Figure 2), despite RDB varied between 63% and 67% (Figure 1b). This was due to the low Fe concentrations in the aerial organs with respect to the roots (Table 2), which accumulated more than 90% of the total Fe content in the plant and suggests the lower contribution of the Fe content in the aerial organs to the development of new tissues. Moreover, the young organs reached increasing accumulations that were much lower than those of the old ones, while in the latter, the values decreased progressively. This trend was also fulfilled in young citrus seedlings [13], although the difference in Fe concentration between the aerial part and the root system was lower (62%), and they also observed a further reduction in absorption in the presence of bicarbonate. In peach plants cultivated in the absence of Fe in the nutrient solution, the stem accumulated the highest concentration of Fe, followed by the leaves, which presented around four times more Fe than the roots [31]. When grown in the presence of Fe, both values were equal and even exceeded those of the stem.

### 2.4. Concentration of ^57^Fe

All the organs of the trees extracted during the cycle were progressively enriched by ^57^Fe (Table 4), as a result of the continuous supply of the isotope from March to December, except for the reproductive organs. Although the flowers/fruits maintained the pattern of Fe accumulation between May and October (111.0%), the maximum increase between samples occurred in the first period (56.7% between May and July) and only between 12.6% and 19.6% in the following periods. In addition, a notable decrease in 30.6% was observed in the last period coinciding with maturity.

The explanation is attributed again to dilution effect, as the total Fe content almost doubled from October to December (Table 3), while it hardly increased in most of the organs during this period. To our knowledge, there are no studies that uses ^57^Fe isotope dilution to determine the seasonal distribution of Fe in citrus that can be discussed. However, fortunately, there is literature that uses the labeling of plants with other isotopes, although the nature and mobility of the mineral element studied must always be taken into account. Thus, in studies on nitrogen absorption, the upward trend was maintained but the increase in the first stages of the plant cycle was very gentle, reached a plateau, and then sharply increased during fruit growth [32]. This uptake pattern is a consequence of the finite capacity of trees to use available soil elements and the capacity to self-regulate net uptake once achieving capacity [32]. By contrast, in some works carried out with ^26^Mg and ^44^Ca [33,34], the authors found lower Mg tracer recovery in roots compared to Ca, due to dilution of root ^26^Mg and internal cycling of Mg through the phloem sap flow [35]. Mg may undergo a rapid transfer to the aboveground biomass compartment without homogenously labeling the root biomass compartment in comparison to Ca.

In all the extractions, the young organs (flowers/fruits, leaves, and branches of the three flushes) achieved considerably greater enrichment than the old ones. The root system (coarse and fibrous roots) showed the least enrichment, again mainly due to the isotope dilution effect that reached the highest total Fe content (Table 3). On the other hand, an increasing gradient in the ^57^Fe enrichments was observed from the coarse root and woody organs to the young organs in the aerial part, which evidences the transport of the isotope from the root system to these sinks. So, the highest concentrations of ^57^Fe of 32.8% and 29.5% were reached in the leaves and branches, respectively, of the fall flush of the plants extracted in December, which were the youngest organs in the entire cycle.

The differences in the enrichment of the various plant organs are consistent with the results obtained in other studies on ^57^Fe in citrus and other crops [13,28,30,36] or with other stable isotopes such as ^15^N or ^44^Ca also in young citrus plants [10,17,26,37,38] and adult plants [20,22,39,40]. In apple, treatment with stable isotope ^44^Ca quantified calcium partitioning even at cell level and resulted in significant enrichment in the cell wall of the pericarp through new cross-bridges of Ca-pectates, thereby enhancing fruit strength and delaying fruit degradation and bitter pit development [41,42].

### 2.5. Fe Content Absorbed from Fertilizer (Fe Uptake) and Its Relative Distribution (Fe Uptake RD)

The Fe absorbed from the fertilizer (Table 5) allows quantification of the fraction of Fe present in each organ absorbed directly from the applied fertilizer; the rest of the Fe until completing the total Fe content (Table 3) comes from other sources, as 1) Fe available in the soil and 2) Fe reserves in the plant from the previous cycle.

Fe uptake increased progressively in all organs and in the plant throughout the cycle except in the flowers/fruits. Fe uptake in reproductive organs decreased from flowering (May) to fruit growth (August), as a result of the Fe content (Table 3) in a greater proportion than the increase in ^57^Fe concentration (Table 4) during this period.

**Figure 3:**
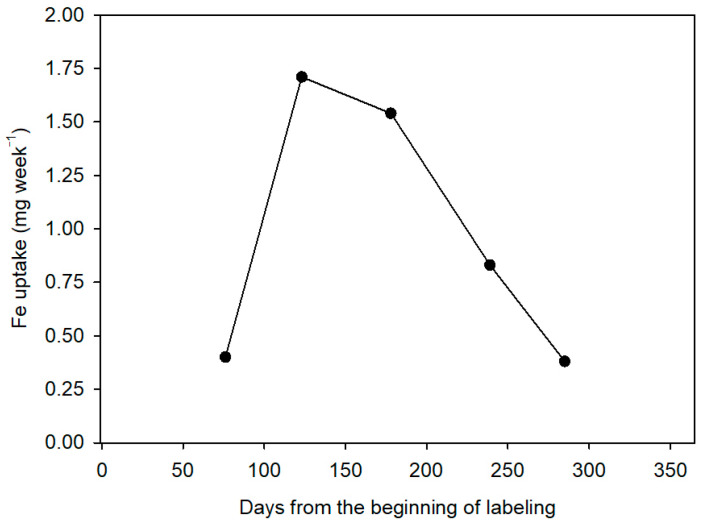
Weekly Fe uptake rate (Fe absorbed from fertilizer week^−1^) measured from the beginning of labeling (March 1) in Nules clementine trees. Each point represents one phenological moment of the plant cycle when plants were uprooted: May (flowering), July (fruit set and the end of fruit drop), August (fruit growth), October (fruit growth), and December (fruit maturity). Values are means of 4 plants.

Figure 3 shows the values of the weekly rate of absorption of Fe, so that in the period of 76 days between the beginning of the vegetative activity (early March) and the end of flowering (middle of May) the plants absorbed about 0.40 mg of Fe per week. The absorption rate increased around fourfold from fruit set and the end of fruit drop (July 1) and stabilized during the phase of rapid fruit growth (August 25). In the third period, from August to October, the absorption rate was reduced by half, and it reached slightly lower values in December than at the beginning of the experiment. The explanation lies in the highest effectiveness of the root system from citrus in taking up the Fe applied in summer in comparison to spring due to two reasons: the higher soil temperatures in summer, which enhances ion uptake [43], and the greater development of fine roots and canopy in the period between the end of fruit drop and fruit maturity (Table 1). Data are in line with other studies on N in citrus that found increased N uptake during the summer months [10,23,37,44,45,46].

**Figure 4:**
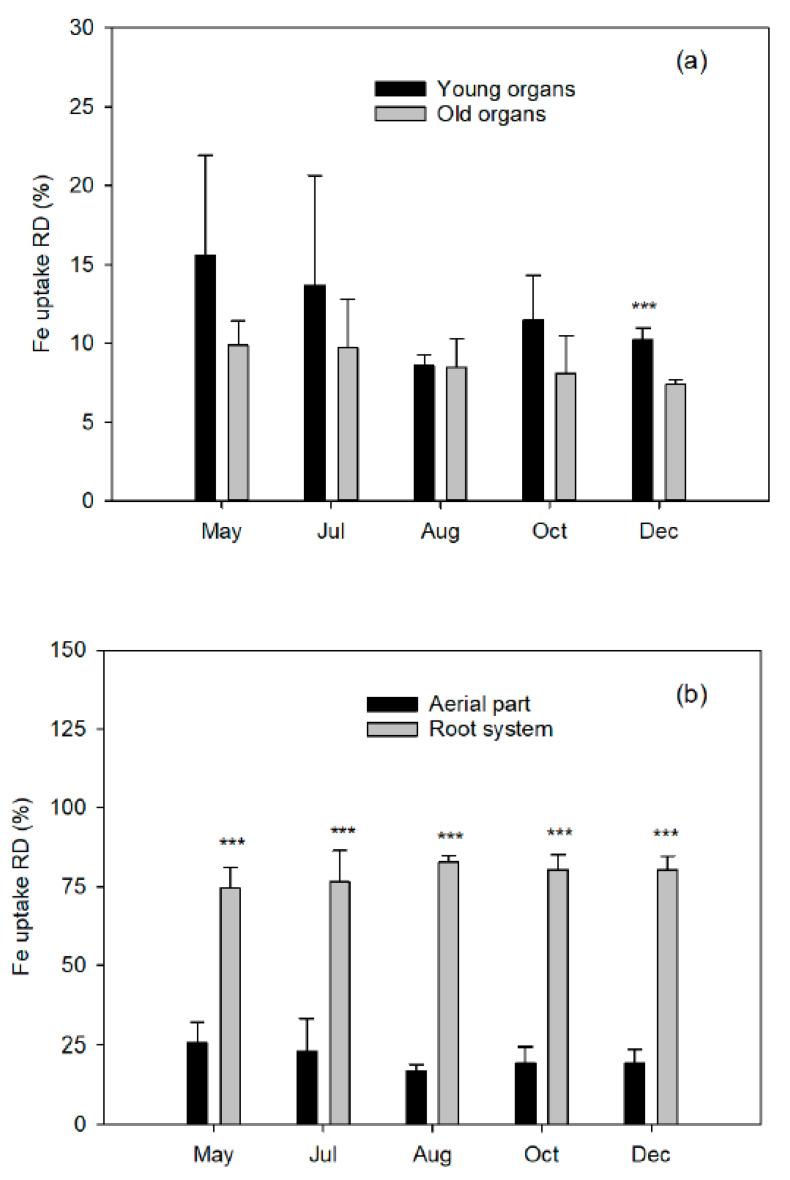
Relative distribution Fe uptake (Fedff^W^) in (**a**) young ^Z^ and old ^Y^ organs from the aerial part in relation to the whole plant and (**b**) aerial part and root system ^X^ of Nules clementine trees uprooted in May (flowering), July (fruit set and the end of fruit drop), August (fruit growth), October (fruit growth), and December (fruit maturity). Values are means ±SD (n = 4). Significant effects of Fe dose are given at *p*  ≤  0.001 (***) at each harvest event. ^Z,Y,X:^ See Figure 1 ^W^RD Fedff (%) organ = ΣFedff (mg) organ × 100/ΣFedff (mg) plant.

Around 80% of the absorbed Fe was accumulated (Fe uptake RD) in the root system and the remaining 20% in the aerial part in all the extractions (Figure 4). This is mainly due to the greater increase in ^57^Fe and biomass enrichment of the fibrous roots observed from May to December (5.3% and 120.3%, respectively) compared to coarse roots (2.7% and 48.5%, respectively).

At this point, the mobility of the element seems to be decisive. Van der Heijden et al. (2013) [33] observed a higher concentration of ^44^Ca in the root system in beech (*Fagus silvatica* L.), associated to the low mobility of Ca and to physiological constraints in the phloem and the higher affinity of Ca for cation exchange sites, such as in the xylem cell wall [47].

Conversely, young orange trees of the Newhall and Valencia cultivars labeled with ^15^N continuously from March to November, resulted on 75% and 61%, respectively, of the N absorbed from the fertilizer when sampling in December [24,48]. These results reflect that the two nutrients are distributed in a completely different way between the organs of the aerial part and the root system [37]. Moreover, Fe uptake by roots and, consequently, the Fe available for translocation is mediated by Fe concentration in the plant [12]. Under conditions of high Fe demand, such as Fe deficiency or probably the appearance of new organs, the plant activates the mechanisms for its absorption through acidification of the cell by proton-ATPase enzyme activity and ferric chelate reductase enzyme, which reduces ferric forms to ferrous forms. That might explain the high Fe uptake RD observed in the root and the elevated enrichment of the sink organs. On the other hand, we should not forget that transport of Fe in the plant is linked to organic acids [49], so a constraint of their synthesis in the Krebs cycle might influence Fe translocation to the aerial part.

Finally, Fe uptake RD among the organs of the aerial part varied according to the time of extraction. Thus, during flowering and fruit set, the young organs accumulated relatively more Fedff than the old ones, even though the FeRD was considerably lower in the young ones (Figure 4). Conversely, at the beginning of August, the Fe uptake RD was distributed almost equally between the two sets of organs. From that moment onwards and until the end of the cycle, it decreased progressively in the old organs, while it followed the opposite trend in the young ones, due to the development of the third flush and the final growth of the fruit.

### 2.6. Percentage of Fe Derived from Fertilizer (Fedff)

The tendency in the percentages of iron derived from fertilizer (Fedff) (Table 6) is identical to the ^57^Fe concentrations (Table 4), since this parameter is calculated as the quotient between the ^57^Fe of each organ or plant and the enrichment of the fertilizer applied (91.80% ^57^Fe). Throughout the successive extractions, the contribution of Fedff to the Fe content of the plant increased considerably as a result of the continuous seasonal input, from near to 2% during the flowering period to 8% in the ripening period.

**Figure 5:**
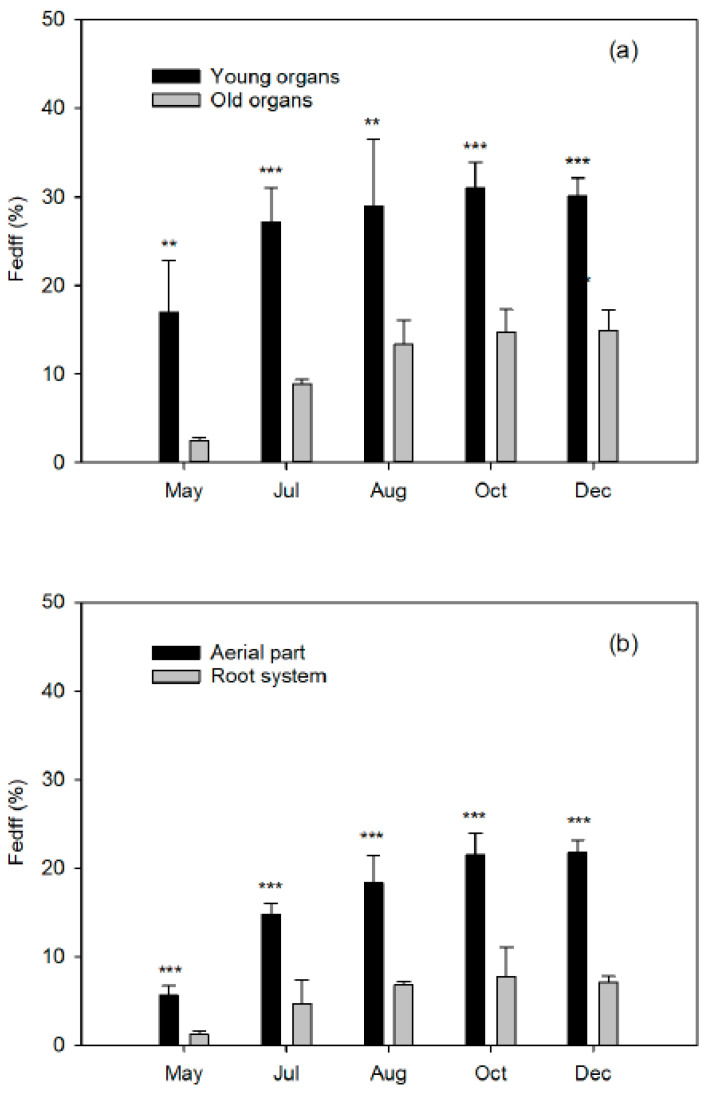
Fe supplied from fertilizer (Fedff^W^) in (**a**) young ^Z^ and old ^Y^ organs from the aerial part in relation to the whole plant and (**b**) aerial part and root system ^X^ of Nules clementine trees uprooted in May (flowering), July (fruit set and the end of fruit drop), August (fruit growth), October (fruit growth), and December (fruit maturity). Values are means ±SD (n = 4). Significant effects of Fe dose are given at *p*  ≤  0.01 (**) and *p*  ≤  0.001 (***) at each harvest event. ^Z,Y,X:^ See Figure 1 ^W^RD Fedff (%) organ = ΣFedff (mg) organ × 100/ΣFedff (mg) plant. ^W^Feddf (%) organ= ^57^Fe (%) organ × 100/91.80% ^57^Fe.

In all the extractions, the values of the Fedff in the young organs (flowers, fruits, and leaves of the developing shoots) were notably higher compared to the old organs although differences decreased as the cycle progressed (Figure 5), from more than sevenfold to barely twice in flowering and the end of the cycle, respectively. This reflects that in the early stages of the vegetative cycle, the Fe supplied with fertilizer preferentially supplies the young organs, which act as sinks. However, it is important to highlight the low net contribution of the applied Fe to the development of the flowers and fruit set, since only 13.8% and 21.4%, respectively, of their Fe content was Fedff and the remaining Fe content up to 100% came from other sources (reserves in woody organs and available in the soil). As the above values are low, it is not necessary to bring forward fertilization or to provide excessive amounts of chelates in early stages of the cycle. This conclusion has also been described for citrus trees in nitrogen fertilization studies. N concentration in new leaves tended to decrease following the growth flushes of March, June, and August [18]. The N derived from fertilizer (%Ndff) was small in April suggesting the importance of internal N reserves for new development in spring. Although Martínez-Alcántara (2012) [10] obtained relative increases along the cycle that were higher than in our study, the lowest increment was produced in flowering (8–13%) compared to increases of 19–27% and 22–41% at the end of the fruit set and fruit growth periods, respectively. The low percentage of Fedff in the flowering period confirms that in early spring, when conditions for root uptake are not conducive, like for N, the course of citrus mainly rely on N remobilization for flower development and spring flush [50]. Moreover, lower N rates led to minor Ndff values in all periods and the diminished Ndff was associated with higher remobilization of stored N, since the amount of N remobilized by young citrus plants depends on external N availability [48]. Therefore, the translocation of Fe from the plant’s reserves during the first months of the cycle seems to be vital for the correct development of both the spring flush and the reproductive organs.

In kiwi, the age of the organ of the aerial part was negatively correlated with the percentage of Fedff when plants were labeled with ^57^Fe for 28 days, and it was maintained even under stress conditions of low Fe levels in the medium [28]. In fact, new leaves from deficient plants were the ones with the highest Fedff recorded (49.2%), with very low levels in the root system (6.8% and 2.25% in fine and coarse roots, respectively). This lends support to our theory of preferential transportation of Fe to the sink organs. However, bicarbonate treatment reduced transport and the highest percentage of Fedff was found in the root, thereby demonstrating the inhibition of the translocation of Fe from root to stem in calcareous environmental conditions.

### 2.7. Use Efficiency of the Applied Fe (FeUE)

This parameter is very useful since the use efficiency of a nutrient indicates the proportion in which the element applied as fertilizer is absorbed by the plant (this can only be quantified with the use of isotopes). However, it should be interpreted with caution because, as it is a relative value Equation (4)), a low efficiency is not always due to a low absorption capacity of the tree, but may be the result of the dose provided exceeding the needs of the crop.

**Figure 6:**
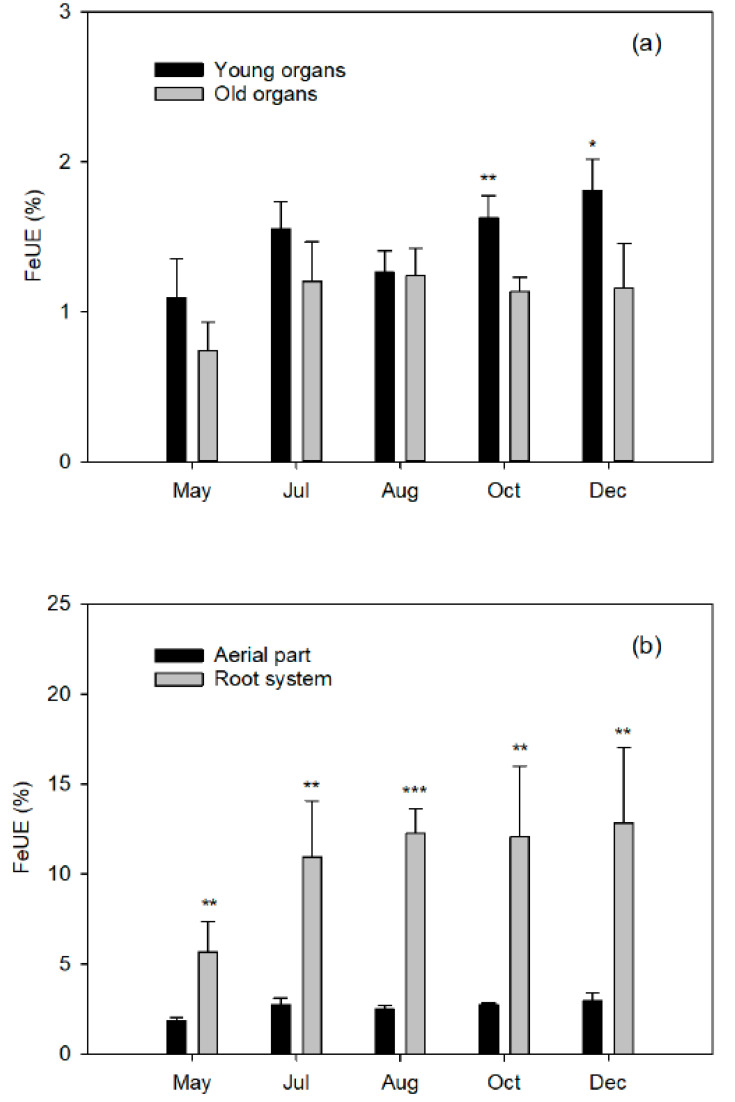
Fe use efficiency (FeUE^W^, %) in (**a**) young ^Z^ and old ^Y^ organs from the aerial part in relation to the whole plant and (**b**) aerial part and root system^X^ of Nules clementine trees uprooted in May (flowering), July (fruit set and the end of fruit drop), August (fruit growth), October (fruit growth), and December (fruit maturity). Values are means ±SD (n = 4). Significant effects of Fe dose are given at *p*  ≤  0.05 (*), *p*  ≤  0.01 (**) and *p*  ≤  0.001 (***) at each harvest event. ^Z,Y,X:^ See Figure 1. ^W^FeUE (%) organ or plant = ΣFeddf (mg) organ or plant × 100 (Table 5)/Fe (mg) applied by fertilizer to every plant from the beginning of the experiment until harvest (Table 7).

In our study, the young organs always reached higher FeUE than the old ones and the highest efficiencies were obtained in the root system (Figure 6). The FeUE of the plant as a whole showed a remarkable increase from mid-May to early July (from 7.52% to 13.72%) and, subsequently, remained constant and barely exceeded 15%. It should be noted that this value is very low and depends on the element in question. The percentages of applied N recovered in plant organs [10] were minimum at flowering (55–25%), while similar ranges were obtained at fruit set (78–35%) and fruit growth (67–36%). Moreover, similar to N, an increasing Fe rate within each phenological period may decrease FeUE, since rates exceeding citrus Fe requirements result in lower FeUE [51,52,53]. Conversely, plants grown under Fe-limiting conditions may have a greater affinity and capacity for Fe uptake due to activation of Strategy I components [12,13] and likely results in higher FeUE.

Low efficiency may be due to two causes: (1) the dose of Fe applied far exceeds the needs for Fe of the plants, which does not seem evident since, according to Table 7, the dose applied is adjusted to the Fe demand of the crop or (2) the soil has a lot of assimilable Fe, which dilutes the ^57^Fe supplied, so plants absorb a higher proportion of the Fe available in the soil than that afforded by the chelate.

## 3. Materials and Methods

### 3.1. Plant Material and Growth Conditions

Four-year-old Nules clementine trees (*Citrus clementina* Hort ex Tan), grafted on Carrizo citrange (*Citrus sinensis* × *Poncirus trifoliata*) rootstock, were grown individually in containers of about 46 l (internal upper and lower diameter of 40 and 45 cm, respectively, and 40 cm high) filled with 65 kg of loamy soil (45% sand, 36% silt, and 19% clay; 27% CaCO_3_; and pH 8.3). The containers were placed outdoors and covered to protect them from the rain. The area was surrounded by a buffer row of identical trees to those used in the experiment. The trial was conducted in the IVIA experimental field station (Moncada, Valencia, Spain; 39°32′ N, 0°23′ W).

Between March (spring growth resumption) and October (end of fruit development) of 2008, 20 trees were fertilized with 240 mg Fe plant^−1^; this rate being consistent with Fe fertilizer guidelines for young citrus trees with a canopy diameter of about 60 cm [53,54] (Legaz et al., 2008). The fertilizer was labeled with ^57^Fe EDDHA, with an abundance of 94.00% (Table 7) in ^57^Fe (Cambridge Isotope Laboratories, Inc., Andover, MA, USA) to discriminate Fe fertilizer from other sources of Fe (especially soil and plant tissues). Table 7 shows the percentage monthly distribution of labeled Fe fertilizer, which was divided into 16 applications and applied every 2 weeks dissolved in a nutrient solution. The rest of the macro- and micronutrients in the nutrient solution were 2 mM N, 0.12 mM P, 0.36 mM K, 0.53 mM Mg, 10 µM Zn, 9 µM Mn, and 0.5 µM Cu, which were provided as KNO_3_, Ca(NO_3_)_2_, H_3_PO_4_, MgSO_4_, Mn chelates, Zn chelates, and CuSO_4_, respectively, as proposed for citrus trees [53,54].

The experiment, therefore, consisted of 20 trees arranged in a randomized block design with five harvests and four replicates of one tree each. The plants were watered using two drip emitters for each tree (4 l h^−1^ emitter^−1^). The amount of water applied to each tree was equivalent to the total seasonal crop evapotranspiration (ETc). The volume of water applied weekly was calculated using the expression: ETc = ETo × Kc, where ETo is the reference crop evapotranspiration under standard conditions, determined using the Penman-Monteith approach [55] and meteorological data from the Agrometeorological Station of the IVIA. Kc (crop coefficient) is a function of canopy size and plantation frame [56]. The soil humidity was monitored daily using a ThetaProbe PR2 (Delta-T Devices, Cambridge, UK), data were acquired by a Moisture meter HH2 (Delta-T Devices Ltd.), and irrigation was scheduled when the matric potential at a depth of 30 cm reached –10 kPa.

The ^57^Fe budget contained in each plant was calculated, and ^57^Fe content in the soil was assumed to be the natural abundance (2.20%) as it was diluted with total Fe and we did not appreciate an increase in ^57^Fe in the soil at the end of the experiment (data not shown).

### 3.2. Plant Uprooting

At five different plant phenological states, the end of flowering (15 May 2008), the end of fruit setting and fruit drop (1 July 2008), two fruit growing moments (1 August and 15 October 2008), and at complete fruit maturity (10 December 2008), a block of four labeled trees were destructively harvested to quantify total dry biomass.

The aerial part was divided into young organs (flowers/fruits, leaves, and twigs of the spring, summer, and fall flushes) and old organs (leaves of the previous year, trunk, and branches). The root system was carefully removed from the soil to avoid tissue loss and divided into coarse and fibrous roots (less than 2 mm diameter). Fresh weights of tree organs were determined, and representative samples of each fraction were taken. All samples were washed in nonionic detergent solution followed by several rinses in deionized water, weighed, frozen in liquid nitrogen, freeze-dried (Telstar LyoAlfa 6, Barcelona, Spain), and powdered in a refrigerated ball mill (IKA A10, Staufen, Germany) after dry-weight determination.

### 3.3. Plant Tissue Analysis

Prior to Fe content analysis, the plant material (0.5 g DW) was calcined in a muffle furnace for 12 h at 550 °C. Subsequently, Fe was extracted with 2% nitric acid (Hiperpur Panreac, Fe < 1 ppb) in an ultrasonic bath (Fungilab S.A., Sant Feliu de Llobregat, Barcelona, Spain) for 30 min at 40 °C and diluted to 50 mL final volume. This extraction was divided into two subsamples to determine total Fe and ^57^Fe concentrations.

Total Fe concentration was measured with the AAS analyzer (iCAP 6000, Thermo Scientific). The abundance in ^57^Fe of each sample was determined using a multiple-collector inductively coupled to an isotope-ratio mass spectrometer (MC-ICP MS, Thermo Finnigan Neptune) [57].

All analyses were carried out with ultrapure water (Ultra Pure Water Systems Milli Q Plus). All determinations were performed in duplicate and a standard was run to ensure accuracy after each set of 10 samples.

### 3.4. Calculations

The ^57^Fe content in each plant compartment was calculated by Equation (1):

^57^Fe_plant compartment_ (µg) = Fe (µg g^−1^) × DW (g) × atom% ^57^Fe excess × 10^−1^ (1)

where atom% ^57^Fe excess was calculated by subtracting the natural abundance of ^57^Fe (2.20%, International Atomic Energy Agency [55]) from the ^57^Fe abundance of each sample.

The content of Fe absorbed by the organ was calculated by Equation (2):

Fe uptake (mg) = Σ[(Fe (%, w/w) × DW (g) × atom% ^57^Fe excess × 10)/(atom% ^57^Fe _fertilizer_)]_plant compartments_. (2)

Iron derived from fertilizer (Fedff) quantifies the contribution of Fe from fertilizer in relation to the total Fe in the organ (Equation (3)):

Fedff (%) = (100 × atom% ^57^Fe excess_plant compartment_)/(atom%^57^Fe _fertilizer_). (3)

Total plant recovery of applied ^57^Fe fertilizer represents the proportion of applied ^57^Fe that is taken up by the tree and embodies its fertilizer Fe use efficiency (FeUE, Equation (4)).

FeUE (%) =100 × ^57^Fe taken up_plant compartment_ (µg)/^57^Fe_fertilizer_ (µg), (4)

where ^57^Fe taken up_plant compartment_ (µg) = Ʃ^57^Fe (µg)_organ_.

### 3.5. Statistical Analysis

Data were subjected to ANOVA, and means were separated by the LSD test at the 5% level to determine the significance of differences among the Fe seasonal distribution within each harvest event, using SAS software (Statistical Analysis System Institute Inc., Cary, NC, USA).

## 4. Conclusions

From the results obtained in the study on the seasonal uptake of Fe in young citrus trees, we conclude that: (1) the biomass in the aerial part was almost twice the amount obtained in the root system. The highest Fe concentrations were achieved in the roots, with the absorbent or fibrous ones accumulating 13 times more Fe than the coarse ones. Therefore, the Fe content in both types of roots accounted for more than 90% of the total amount in the plant. (2) The contribution of applied Fe to the development of flowering and fruit set was only 17% and 27% of the total Fe content. This suggests that the Fe applied in the early stages contributed significantly less to the development of new tissues than that coming from the plant reserves and the soil. Hence, there is no need to bring forward fertilization or to apply excessive amounts of chelates in the abovementioned periods. (3) The applied ^57^Fe was mostly concentrated in the young organs of the aerial part, which acted as a sink for it. Despite this, between these organs and the old ones only about 20% of the total Fe was absorbed by the plant and the rest up to 100% was retained in the root set. (4) The highest rates of seasonal absorption of applied Fe were reached in the periods of fruit growth (beginning of July until the end of August), these being the times when the greatest contributions must be made. (5) The use efficiency of the applied Fe barely exceeded 15%, which reflects that despite the fact that it is a fairly calcareous soil, the supplied ^57^Fe was diluted with assimilable native Fe and the plants absorbed a greater proportion of the available Fe than that provided with the chelate. Finally, (6) as a basic conclusion, it is suggested that the monthly distribution and number of applications of Fe compounds to citrus should be performed following the pattern described in this study, since the seasonal absorption curve showed a very similar response to this one.

## Figures and Tables

**Table 1:** Dry-biomass (g, DW plant^−1^) partitioning among the main organs of Nules clementine trees uprooted in May (flowering), July (fruit set and the end of fruit drop), August (fruit growth), October (fruit growth), and December (fruit maturity). Values are means ± SD (n = 4). Different lowercase letters indicate significant differences based on one-way analysis of variance. Significant effects of the Fe dose applied are given at *p*  ≤  0.01 (**), *p*  ≤  0.001 (***), and ns (not significance) at each plant component. *p*-value between brackets (LSD test). DW: dry weight.

	May				Jul				Aug				Oct				Dec				ANOVA
Flowers/fruits ^Z^	6.8	±	3.0	c	5.3		2.1	c	8.5	±	0.6	c	28.0	±	4.7	b	60.2	±	7.0	a	*** (0.0000)
Autumn leaves													54.9	±	8.5		56.3	±	13.7		ns (0.8674)
Summer leaves					71.6	±	35.4		88.9	±	62.3		91.3	±	37.3		92.4	±	32.4		ns (0.8947)
Spring leaves	84.4	±	24.1		95.9	±	33.5		93.1	±	24.1		114.7	±	10.5		125.5	±	10.3		ns (0.1096)
Autumn branches													12.2	±	7.1		14.5	±	3.0		ns (0.57658)
Summer branches					9.6	±	3.9	c	15.6	±	5.2	bc	23.5	±	7.1	a	22.5	±	3.8	ab	** (0.0078)
Spring branches	13.7	±	3.8	b	17.5	±	5.6	b	25.7	±	5.1	a	25.1	±	1.6	a	27.0	±	4.0	a	** (0.0014)
Old leaves	115.3	±	35.3		110.0	±	36.5		96.2	±	28.5		87.4	±	31.3		79.8	±	6.9		ns (0.4346)
Old branches	122.1	±	15.2	c	152.4	±	32.1	bc	177.3	±	39.9	ab	186.3	±	7.7	ab	200.4	±	8.7	a	** (0.0033)
Trunk	124.9	±	11.9	c	156.7	±	17.5	b	175.4	±	3.7	b	210.4	±	37.9	a	220.0	±	12.6	a	*** (0.0000)
Coarse root	194.7	±	45.3		200.6	±	41.3		219.5	±	41.4		270.6	±	142.3		289.2	±	134.2		ns (0.5272)
Fibrous root	84.1	±	10.7	c	109.8	±	7.8	c	149.2	±	6.4	b	164.1	±	29.5	ab	185.3	±	36.7	a	*** (0.0001)
Total plant	746.0	±	92.4	c	929.5	±	97.7	b	1049.4	±	113.2	b	1268.6	±	184.4	a	1373.1	±	182.5	a	*** (0.0000)

^Z^ Fruits/flowers, in May: flowers, floral button and ovaries; Jul, end of fruit set; Aug and Oct: developing fruits; and, Dec: mature fruits.

**Table 2:** Total Fe concentration (mg Fe 1000 gDW^−1^) in the main organs and ponderated Fe concentration (FePC)^Y^ of Nules clementine trees uprooted in May (flowering), July (fruit set and the end of fruit drop), August (fruit growth), October (fruit growth), and December (fruit maturity). Values are means ± SD (n = 4). Different lowercase letters indicate significant differences based on one-way analysis of variance. Significant effects of the Fe dose applied are given at *p*  ≤  0.001 (***) and ns (not significance) at each plant component. *p*-value between brackets (LSD test). DW: dry weight.

	May				Jul				Aug				Oct				Dec				ANOVA
Flowers/fruits ^Z^	52.2	±	2.1	a	36.0	±	8.8	b	16.1	±	2.6	c	13.0	±	1.0	c	11.1	±	0.8	c	*** (0.0000)
Autumn leaves													27.1	±	5.4		31.2	±	2.5		ns (0.2183)
Summer leaves					34.4	±	1.9		37.2	±	1.7		40.9	±	6.1		41.0	±	5.0		ns (0.1208)
Spring leaves	34.3	±	9.4		36.1	±	3.7		39.1	±	2.7		41.8	±	6.1		42.3	±	3.9		ns (0.2526)
Autumn branches													26.1	±	6.2		24.2	±	2.2		ns (0.5892)
Summer branches					27.4	±	2.8		30.3	±	4.9		31.1	±	5.2		32.2	±	2.3		ns (0.4032)
Spring branches	29.2	±	2.4		36.3	±	5.4		39.2	±	3.2		39.2	±	6.0		39.2	±	7.5		ns (0.0681)
Old leaves	59.2	±	3.6		45.4	±	7.9		50.2	±	9.0		48.3	±	8.1		49.7	±	8.7		ns (0.1803)
Old branches	37.1	±	2.8		34.5	±	5.0		36.2	±	2.1		36.4	±	4.0		34.5	±	6.1		ns (0.8657)
Trunk	45.1	±	7.6		41.3	±	4.7		38.1	±	2.9		38.5	±	6.8		37.0	±	8.2		ns (0.4091)
Coarse root	171.4	±	11.1	b	223.6	±	18.9	a	209.3	±	22.6	a	173.0	±	16.1	b	163.6	±	25.6	b	*** (0.0017)
Fibrous root	2391.2	±	327.2	b	2877.4	±	327.5	a	2406.1	±	360.2	b	2350.7	±	287.3	b	2118.2	±	173.3	b	*** (0.0379)
Total plant ^Y^	345.3	±	59.4		415.5	±	49.9		411.1	±	42.7		376.5	±	56.5		343.1	±	39.6		ns (0.1626)

^Z^ Fruits/flowers, in May: flowers, floral button and ovaries; Jul, end of fruit set; Aug and Oct: developing fruits; and, Dec: mature fruits. ^Y^ FePC: (mg Fe/1000 g DW) plant =ΣFe content (mg) plant × 1000/ΣDW(g) plant.

**Table 3:** Total Fe content (mg Fe) in the main organs and the plant of Nules clementine trees uprooted in May (flowering), July (fruit set and the end of fruit drop), August (fruit growth), October (fruit growth), and December (fruit maturity). Values are means ± SD (n = 4). Different lowercase letters indicate significant differences based on one-way analysis of variance. Significant effects of the Fe dose applied are given at *p*  ≤  0.05 (*), *p*  ≤  0.01 (**), *p*  ≤  0.001 (***), and ns (not significance) at each plant component. *p*-value between brackets (LSD test). DW: dry weight.

	May				Jul				Aug				Oct				Dec				ANOVA
Flowers/fruits ^Z^	0.4	±	0.2	b	0.2	±	0.1	c	0.1	±	0.0	c	0.4	±	0.1	b	0.7	±	0.1	a	*** (0.0000)
Autumn leaves													1.5	±	0.5		1.8	±	0.5		ns (0.4915)
Summer leaves					2.5	±	1.2		3.3	±	2.4		3.9	±	1.9		3.9	±	1.6		ns (0.6614)
Spring leaves	2.9	±	1.0	c	3.4	±	1.0	bc	3.7	±	1.1	bc	4.8	±	0.4	ab	5.3	±	0.9	a	** (0.0084)
Autumn branches													0.3	±	0.1		0.4	±	0.1		ns (0.4782)
Summer branches					0.3	±	0.1	b	0.5	±	0.2	ab	0.7	±	0.1	a	0.7	±	0.2	a	** (0.0045)
Spring branches	0.4	±	0.1	b	0.6	±	0.2	b	1.0	±	0.1	a	1.0	±	0.2	a	1.1	±	0.3	a	** (0.0014)
Old leaves	6.7	±	1.7		5.2	±	2.3		5.0	±	2.1		4.1	±	1.2		4.0	±	0.7		ns (0.2080)
Old branches	4.5	±	0.7	b	5.2	±	1.1	ab	6.5	±	1.7	a	6.8	±	0.8	a	6.9	±	1.2	a	* (0.0421)
Trunk	5.7	±	1.2	b	6.5	±	1.4	ab	6.7	±	0.4	ab	7.9	±	0.7	a	8.1	±	1.5	a	* (0.0374)
Coarse root	33.5	±	8.7		45.3	±	13.1		46.4	±	12.6		47.6	±	26.1		46.1	±	20.6		ns (0.7751)
Fibrous root	202.7	±	44.6	b	317.6	±	56.1	a	360.7	±	69.1	a	383.0	±	70.9	a	392.8	±	86.2	a	** (0.0069)
Total plant ^Y^	256.8	±	42.0	b	386.7	±	59.4	a	433.8	±	84.5	a	461.9	±	81.6	a	471.7	±	107.4	a	*** (0.0090)

^Z^ Fruits/flowers, in May: flowers, floral button and ovaries; Jul, end of fruit set; Aug and Oct: developing fruits; and, Dec: mature fruits. Fe (mg) organ = organ Fe concentration (%) × organ DW (mg). ^Y^ Fe (mg) plant = ΣFe (mg) organ in the plant.

**Table 4:** Labeled Fe enrichment (E^57^Fe, atom% ^57^Fe excess) in the main organs and ^57^Fe ponderated enrichment in the plant (^57^Fe PE ^Y^) of Nules clementine trees uprooted in May (flowering), July (fruit set and the end of fruit drop), August (fruit growth), October (fruit growth), and December (fruit maturity). Values are means ± SD (n = 4). Different lowercase letters indicate significant differences based on one-way analysis of variance. Significant effects of the Fe dose applied are given at *p*  ≤  0.05 (*), *p*  ≤  0.001 (***), and ns (not significance) at each plant component. *p*-value between brackets (LSD test).

	May				Jul				Aug				Oct				Dec				ANOVA
Flowers/fruits ^Z^	12.7	±	2.4	c	19.9	±	3.5	b	22.4	±	4.4	ab	26.8	±	2.8	a	18.6	±	2.2	b	*** (0.0003)
Autumn leaves													31.8	±	4.3		32.8	±	3.8		ns (0.7371)
Summer leaves					28.9	±	3.6		29.8	±	5.5		30.6	±	4.6		31.7	±	4.2		ns (0.8408)
Spring leaves	16.9	±	5.9		23.4	±	7.1		25.4	±	13.5		28.8	±	4.6		29.5	±	2.6		ns(0.1900)
Autumn branches													25.0	±	5.0		25.4	±	2.1		ns (0.8955)
Summer branches					23.3	±	1.3		24.3	±	4.5		24.4	±	4.3		24.3	±	4.8		ns (0.9772)
Spring branches	9.1	±	6.6	b	15.3	±	5.8	ab	18.7	±	2.6	a	19.0	±	2.2	a	20.3	±	3.9	a	* (0.0222)
Old leaves	1.7	±	0.6	c	6.5	±	2.6	b	8.3	±	1.8	ab	10.0	±	2.6	a	10.3	±	3.3	a	*** (0.0007)
Old branches	2.4	±	1.7	b	11.5	±	1.2	a	16.0	±	3.7	a	16.0	±	4.5	a	15.9	±	3.5	a	*** (0.0001)
Trunk	1.4	±	1.0	c	6.6	±	1.3	b	11.0	±	2.6	a	12.4	±	2.6	a	12.9	±	3.2	a	*** (0.0000)
Coarse root	0.6	±	0.2	b	0.9	±	0.4	b	2.5	±	0.6	a	3.1	±	1.5	a	3.3	±	0.7	a	*** (0.0005)
Fibrous root	1.4	±	0.8	c	3.5	±	1.5	b	5.6	±	0.7	a	6.4	±	2.0	a	6.7	±	1.5	a	*** (0.0003)
Total plant ^Y^	1.6	±	0.3	d	3.8	±	0.3	c	6.0	±	0.8	b	6.9	±	0.8	a	7.3	±	0.6	a	*** (0.0000)

^Z^ Fruits/flowers, in May: flowers, floral button and ovaries; Jul, end of fruit set; Aug and Oct: developing fruits; and, Dec: mature fruits. E^57^Fe (%) organ= ^57^Fe (%) organ – 2.20 % ^57^Fe (natural abundance in the isotope). ^57^Fe PE (%) plant = Σ^57^Fe (mg) organs plant × 100/ΣFe (mg) organs plant, where ^57^Fe (mg) organ = ^57^Fe (%) organ × Fe content (mg) organ.

**Table 5:** ^57^Fe uptake (mg ^57^Fe) in the main organs of Nules clementine trees uprooted in May (flowering), July (fruit set and the end of fruit drop), August (fruit growth), October (fruit growth), and December (fruit maturity). Values are means ± SD (n = 4). Different lowercase letters indicate significant differences based on one-way analysis of variance. Significant effects of the Fe dose applied are given at *p*  ≤  0.05 (*), *p*  ≤  0.01 (**), *p*  ≤  0.001 (***), and ns (not significance) at each plant component. *p*-value between brackets (LSD test).

	May				Jul				Aug				Oct				Dec				ANOVA
Flowers/fruits ^Z^	0.05	±	0.01	c	0.04	±	0.02	c	0.03	±	0.01	c	0.10	±	0.02	b	0.13	±	0.01	a	***(0.0000)
Autumn leaves													0.53	±	0.18		0.63	±	0.23		ns(0.4888)
Summer leaves					0.76	±	0.35		0.98	±	0.69		1.22	±	0.49		1.34	±	0.64		ns(0.4955)
Spring leaves	0.50	±	0.15	c	0.90	±	0.47	bc	1.00	±	0.63	bc	1.48	±	0.18	ab	1.71	±	0.27	a	**(0.0037)
Autumn branches													0.08	±	0.05		0.10	±	0.02		ns(0.6066)
Summer branches					0.07	±	0.02		0.14	±	0.09		0.19	±	0.06		0.20	±	0.08		ns(0.0661)
Spring branches	0.05	±	0.01	b	0.10	±	0.02	b	0.21	±	0.05	a	0.20	±	0.03	a	0.23	±	0.07	a	***(0.0001)
Old leaves	0.1	±	0.07	b	0.32	±	0.11	ab	0.44	±	0.19	a	0.44	±	0.16	a	0.45	±	0.17	a	*(0.0305)
Old branches	0.2	±	0.02	c	0.65	±	0.14	b	1.08	±	0.13	a	1.15	±	0.18	a	1.22	±	0.44	a	***(0.0000)
Trunk	0.1	±	0.02	d	0.47	±	0.13	c	0.79	±	0.19	b	1.07	±	0.22	ab	1.12	±	0.28	a	***(0.0000)
Coarse root	0.2	±	0.07	b	0.44	±	0.15	b	1.22	±	0.24	a	1.58	±	0.69	a	1.71	±	0.88	a	**(0.0023)
Fibrous root	2.9	±	0.88	c	12.71	±	7.17	bc	21.59	±	2.34	ab	26.65	±	8.62	a	29.08	±	9.18	a	***(0.0003)
Total plant ^Y^	4.1	±	0.91	d	16.47	±	7.45	c	27.48	±	2.55	b	34.69	±	9.29	ab	37.92	±	10.88	a	***(0.0000)

^Z^ Fruits/flowers, in May: flowers, floral button and ovaries; Jul, end of fruit set; Aug and Oct: developing fruits; and, Dec: mature fruits. Fe uptake (mg) organ = ^57^Fe (mg) organ × 100/91.80 % ^57^Fe (applied in the fertilizer), where ^57^Fe (mg) organ = ^57^Fe (%) organ x Fe content (mg) organ. ^Y^ Fe uptake (mg) plant = ΣFe uptake (mg) in all the organs from the plant.

**Table 6:** Fe derived from fertilizer (Fedff, mg Fe) in the main organs of Nules clementine trees uprooted in May (flowering), July (fruit set and the end of fruit drop), August (fruit growth), October (fruit growth), and December (fruit maturity). Values are means ± SD (n = 4). Different lowercase letters indicate significant differences based on one-way analysis of variance. Significant effects of the Fe dose applied are given at *p*  ≤  0.001 (***) and ns (not significance) at each plant component. *p*-value between brackets (LSD test).

	May				Jul				Aug				Oct				Dec				ANOVA
Flowers/fruits ^Z^	13.8	±	2.6	c	21.7	±	3.8	b	24.4	±	4.8	ab	29.1	±	3.0	a	20.2	±	2.4	a	*** (0.0003)
Autumn leaves													34.6	±	4.6		35.7	±	4.1		ns (0.7374)
Summer leaves					31.4	±	3.9		32.5	±	6.0		33.3	±	5.0		34.5	±	4.6		ns (0.8412)
Spring leaves	18.4	±	6.4		25.5	±	7.7		27.7	±	14.7		31.4	±	5.0		32.2	±	2.8		ns (0.1899)
Autumn branches													27.2	±	5.5		27.6	±	2.2		ns (0.8957)
Summer branches					25.4	±	1.4		26.5	±	4.9		26.6	±	4.7		26.5	±	5.2		ns (0.9772)
Spring branches	13.1	±	2.8	b	16.7	±	6.4	ab	20.4	±	2.8	a	20.7	±	2.4	a	22.1	±	4.2	a	*** (0.1385)
Old leaves	1.9	±	0.6	c	7.0	±	2.8	b	9.1	±	1.9	ab	10.9	±	2.8	ab	11.2	±	3.6	a	*** (0.0007)
Old branches	3.4	±	0.6	b	12.5	±	1.3	a	17.4	±	4.0	a	17.4	±	4.9	a	17.3	±	3.8	a	*** (0.0001)
Trunk	2.1	±	0.1	c	7.2	±	1.4	b	11.9	±	2.9	a	13.5	±	2.8	a	14.1	±	3.5	a	*** (0.0000)
Coarse root	0.6	±	0.2	b	1.0	±	0.4	b	2.7	±	0.6	a	3.4	±	1.7	a	3.6	±	0.8	a	*** (0.0005)
Fibrous root	1.5	±	0.8	c	3.8	±	1.6	b	6.1	±	0.8	a	7.0	±	2.2	a	7.3	±	1.6	a	*** (0.0003)
Total plant ^Y^	1.8	±	0.2	d	4.1	±	0.3	c	6.5	±	0.9	b	7.6	±	0.9	a	8.0	±	0.6	a	*** (0.0000)

^Z^ Fruits/flowers, in May: flowers, floral button and ovaries; Jul, end of fruit set; Aug and Oct: developing fruits; and, Dec: mature fruits. Fe ddf (%) organ or plant = ^57^Fe (%) organ or plant × 100/91.80% ^57^Fe (in the applied fertilizer).

**Table 7:** Fe and ^57^Fe doses and its monthly distribution along the fertilizing period in citrus trees.

	Mar	Apr	May	Jun	Jul	Aug	Sep	Oct	Total
Fe distribution rate (%)	5	10	15	20	20	15	10	5	100
Fe dose (mg plant^−1^) ^Z^	12	24	36	48	48	36	24	12	240
^57^Fe dose (mg plant^−^^1^) ^Y^	11.28	22.56	33.84	44.12	44.14	33.84	22.56	11.28	220.6

^Z^ Total Fe supplied per plant. ^Y^ Labeled Fe supplied per plant (mg ^57^Fe plant^−1^). Fertilizer was supplied as labeled chelate enriched at 94% with the stable isotope ^57^Fe.
